# Bloody Zebrafish: Novel Methods in Normal and Malignant Hematopoiesis

**DOI:** 10.3389/fcell.2018.00124

**Published:** 2018-10-15

**Authors:** Emma de Pater, Eirini Trompouki

**Affiliations:** ^1^Department of Hematology, Erasmus MC, Rotterdam, Netherlands; ^2^Department of Cellular and Molecular Immunology, Max Planck Institute of Immunobiology and Epigenetics, Freiburg im Breisgau, Germany

**Keywords:** zebrafish, hematopoiesis, next generation sequencing, hematopoietic (stem) cells, technology

## Abstract

Hematopoiesis is an optimal system for studying stem cell maintenance and lineage differentiation under physiological and pathological conditions. In vertebrate organisms, billions of differentiated hematopoietic cells need to be continuously produced to replenish the blood cell pool. Disruptions in this process have immediate consequences for oxygen transport, responses against pathogens, maintenance of hemostasis and vascular integrity. Zebrafish is a widely used and well-established model for studying the hematopoietic system. Several new hematopoietic regulators were identified in genetic and chemical screens using the zebrafish model. Moreover, zebrafish enables *in vivo* imaging of hematopoietic stem cell generation and differentiation during embryogenesis, and adulthood. Finally, zebrafish has been used to model hematopoietic diseases. Recent technological advances in single-cell transcriptome analysis, epigenetic regulation, proteomics, metabolomics, and processing of large data sets promise to transform the current understanding of normal, abnormal, and malignant hematopoiesis. In this perspective, we discuss how the zebrafish model has proven beneficial for studying physiological and pathological hematopoiesis and how these novel technologies are transforming the field.

## Introduction

Over the past three decades, zebrafish has been established as an important model to study various biological processes during development and homeostasis, including hematopoiesis. Many attractive features underpin the success of zebrafish as a model for vertebrate hematopoiesis. Cell-intrinsic and -extrinsic signaling mechanisms in hematopoiesis are well conserved between zebrafish and mammals, with the exception of a few hematopoietic niche components (Liao et al., 1998; Murayama et al., 2006; Bertrand and Traver, 2009; Paik and Zon, 2010; Goessling and North, 2011; Zhang and Liu, 2011; Zhang et al., 2013; Frame et al., 2017; Nik et al., 2017; Gore et al., 2018). Moreover, zebrafish embryos are small and transparent so they are ideal for imaging and easy to manipulate, at low cost. Additionally, genetic manipulation is easy and population studies can be easily performed in zebrafish. Thus, zebrafish have become invaluable vertebrate models for robust large-scale genetic screens (Mullins et al., 1994; Driever et al., 1996; Amsterdam et al., 1999) and, more recently, high-throughput chemical compound screens (North et al., 2007; Yeh et al., 2009). However, there are certain disadvantages in the zebrafish model. For example, zebrafish is not a mammal, but rather a poikilothermic animal in which the development of embryos occurs outside of the animal body and without placenta. That may lead to many metabolic and other differences between zebrafish and mammals, including drug action and utilization. Finally, the zebrafish genome is duplicated and thus many genes have paralogs and homologs that make the otherwise easy genetic manipulation complicated (Glasauer and Neuhauss, 2014).

**FIGURE 1:**
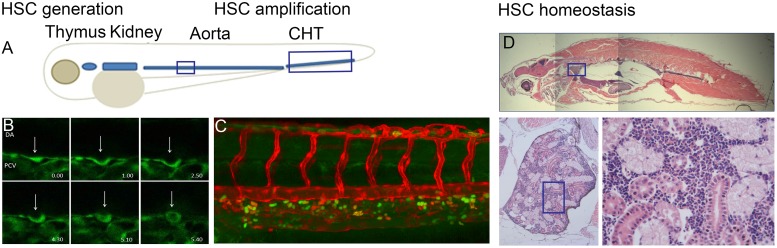
**(A)** Overview of definitive hematopoietic sites in the developing embryo where HSPCs are born from hemogenic endothelial cells of the dorsal aorta (DA). HSPCs are amplified in the caudal hematopoietic tissue (CHT) and migrate to the kidney and thymus. **(B)** Endothelial-to-hematopoietic transition (EHT) event imaged in a Tg(fli:GFP) embryo between 32 and 40 hpf. **(C)** CHT region in Tg(flt:RFP)/Tg(CD41:GFP) embryo at 56 hpf indicating erythroid myeloid progenitors (EMP) in green and definitive HSPCs in yellow as they originate from the artery and retain RFP at this timepoint. **(D)** Sagittal section through an adult zebrafish where the head kidney is indicated with enlargement showing the hematopoietic cells in between the kidney tubules.

Embryonic hematopoiesis in zebrafish is a multistep process occurring in a spatially restricted manner in three distinct waves. During the intraembryonic primitive wave, the medial and anterior lateral mesoderm give rise to erythroid and myeloid cells, respectively. Erythro-myeloid progenitors (EMPs) form in the posterior blood island (PBI) during a transient intermediate wave. Finally, during the definitive wave, hematopoietic stem cells (HSC) with multilineage capacity originate in the aorta-gonad-mesonephros (AGM) region. The HSCs then translocate to and expand in the caudal hematopoietic tissue (CHT), which is followed by the colonization of the kidney and the thymus (**Figure 1**). Interestingly, it was recently discovered that HSC-independent T-cells can originate from the AGM and PBI during the embryonic and larval stages of development (Tian et al., 2017).

Zebrafish has been extensively used for modeling human hematopoietic disease, including anemia, thrombocytopenia, bone marrow failure syndromes, leukemia, and lymphoma (Taylor and Zon, 2011; Kwan and North, 2017; Potts and Bowman, 2017; Gore et al., 2018). The first transplantable leukemia modeled in zebrafish was T-cell acute lymphoblastic leukemia (T-ALL), which was induced by T cell-specific c-Myc overexpression (Langenau et al., 2003). Thereafter, several models of myelodysplastic syndromes and myeloproliferative neoplasms have been described (Le et al., 2007; He et al., 2014; Gjini et al., 2015; Peng et al., 2015).

Although for many years zebrafish was mainly used to study embryonic and larval developmental hematopoiesis, recent technological advances have transformed the field. In this perspective, we will briefly discuss how research using zebrafish genetic models in combination with chemical screens, high-end imaging, and genome-wide molecular, metabolics and proteomics approaches has contributed to our understanding of hematopoiesis.

## Hematopoietic Generation, Lineage Tracing, and Differentiation

### Imaging the Origin of Hematopoiesis

The transparency and accessibility of zebrafish embryos was pivotal to collect evidence showing that hematopoietic stem and progenitor cells (HSPCs) emerge from the ventral wall of the dorsal aorta *in vivo* (Bertrand et al., 2010; Kissa and Herbomel, 2010). Moreover, high-end imaging techniques in zebrafish embryos uncovered the mechanisms of thymus development (Hess and Boehm, 2012) and revealed that HSPCs are amplified and interact with endothelial cells in the CHT (Tamplin et al., 2015). Multiple signaling pathways and cell-interactions affect HSPC emergence. For instance, inflammatory signaling provided by neutrophils is required for HSC generation (Espin-Palazon et al., 2014; Li et al., 2014; Sawamiphak et al., 2014; He et al., 2015). These unique properties of zebrafish allowed to uncover the role of macrophages in HPSC mobilization and definitive hematopoiesis (Travnickova et al., 2015). Where transient and rapid cell-interactions occur, light-sheet microscopy, SPIM (selective plane illumination) or spinning disk microscopy can be used to visualize these processes *in vivo* in embryos and adults, because these systems record time-lapse 3D fluorescent images 100–1000x faster than conventional confocal microscopy (Inoue and Inoue, 1996; Huisken et al., 2004; Arrenberg et al., 2010). Moreover, transparent adult zebrafish models (White et al., 2008) have enabled the imaging of adult hematopoiesis, thereby opening the way to research exploring different HSC niche components and HSC-niche interactions in the adult kidney marrow, thymus, and spleen.

### Lineage Differentiation: The Impact of Single-Cell RNA Sequencing

The development of single-cell RNA-sequencing (scRNA-seq) revolutionized the way we understand hematopoiesis. As most cellular compartments have a certain degree of heterogeneity, with bulk RNA-seq one cannot distinguish between a small transcriptional difference in many cells, and a large transcriptional difference in a few cells. Several insightful reviews describe the different methods used for single-cell RNA-seq (Kolodziejczyk et al., 2015; Ziegenhain et al., 2017; Dal Molin and Di Camillo, 2018).

In zebrafish, one of the first methods used to characterize the transcriptome of single cells was massive parallel qPCR, where up to 96 transcripts could be analyzed in great sequencing depth using the Fluidigm system. This method revealed two distinct sub-populations of HSPCs in the CD41-GFP low-expressing stem cell compartment of the adult kidney marrow. Moreover, by using this technique and genetic ablation of T cells, a previously uncharacterized hematopoietic cytotoxic T/NK cell population in zebrafish was uncovered (Moore et al., 2016).

Recent technological advances in scRNA-seq have enabled analyses without restriction to specific transcripts. A re-examination of the CD41-GFP^low^ population revealed four HSPC sub-populations with different cellular characteristics and potential novel markers for HSCs were uncovered. Importantly, some cells in these subpopulations expressed the thrombocyte differentiation program long before they would have been characterized as thrombocytes, showing that there is an early lineage bias (Guo et al., 2013; Buenrostro et al., 2015; Paul et al., 2015; Drissen et al., 2016; Grover et al., 2016; Nestorowa et al., 2016; Olsson et al., 2016; Alberti-Servera et al., 2017; Velten et al., 2017; Villani et al., 2017; Buenrostro et al., 2018; Dahlin et al., 2018). In addition, scRNA-seq analyses in various transgenic lines revealed that ribosomal genes and lineage regulators control hematopoietic differentiation (Athanasiadis et al., 2017) and uncovered several novel hematopoietic populations, including two new types of NK cells (Tang et al., 2017). Finally, elegant comparative evolutionary studies on LCK-GFP transgenic zebrafish and mammals showed that membrane proteins are less conserved in NK cells than in T cells (Carmona et al., 2017). In the pathological context, scRNA-seq analysis of Myc-induced T-ALLs demonstrated that few cells expressed an immature stem cell program, suggesting that only a small proportion of leukemia cells promote the disease. This is remarkable as a single transgenic approach was used to initiate leukemogenesis and all leukemia cells overexpress Myc (Moore et al., 2016).

### Lineage Tracing

Zebrafish has traditionally been utilized to lineage-trace differentiation during embryonic stages by labeling single cells with dyes and following them throughout development, until the dye fades or dilutes. However, the recent development of complex genetic models has removed this time restriction and enabled lineage tracing from the embryo into adulthood. For instance, HSPCs generated from the hemogenic endothelium of the aorta have been lineage-traced by using the multicolor transgenic labeling system “blood bow” (Henninger et al., 2017) in combination with high-end imaging and fluorescence-activated cell sorting (FACS). Additionally, labeling with CRISPR/Cas9 scarring in embryos and tracing of unique hematopoietic clones into adulthood has revealed that the hematopoietic system is only generated from a handful of cells present at dome stage (Alemany et al., 2018). This study claimed that all clones contribute to all blood lineages, a subject that is controversial in mammalian studies (Yamamoto et al., 2013; Notta et al., 2016; Pei et al., 2017).

A different approach for lineage-tracing cells consists of performing high-throughput scRNAseq at various developmental stages and then mapping similarities in transcriptional profiles across a pseudo timescale of differentiation (Macosko et al., 2015). By using this method in early embryogenesis, two independent studies have described gradually divergent differentiation patterns for specific lineages and uncovered signaling networks required for zebrafish development (Farrell et al., 2018; Wagner et al., 2018).

Future studies combining scRNAseq with lineage tracing will be paramount to advance our understanding of the developmental origins of hematopoietic populations. However, this approach has the important caveat that scRNA-seq does not provide topographic information for each individual cell. To overcome this limitation, the Van Oudernaarden and Bakkers laboratories have developed RNA-tomography (TOMOSEQ), a method that combines traditional histological techniques with low-input RNA sequencing and mathematical image reconstruction (Junker et al., 2014).

## Identification of Novel Regulatory Mechanisms of Normal and Malignant Hematopoiesis

### Chemical Screens to Identify Regulators of Normal and Abnormal Hematopoiesis

Zebrafish is an ideal vertebrate model system to conduct bio-reactive compound screens (Zon and Peterson, 2005; Cusick et al., 2012; Tamplin et al., 2012; Veinotte et al., 2014; Rennekamp and Peterson, 2015; Deveau et al., 2017). The animals are small-sized and lay hundreds of eggs that develop very rapidly, thereby allowing the monitoring of compound activity and biotoxicity *in vivo* across development. Such screens have led to the identification of prostaglandin E2 as a compound that increases HSC production (North et al., 2007). Prostaglandin E2 is currently being investigated for HSC expansion applications in human and non-human primates (Goessling et al., 2011; Cutler et al., 2013).

Important insights into the molecular regulation of T-ALL came from zebrafish studies where immature T cells served as models for T-ALL cells. By screening small molecules for an effect on immature T cells using LCK-GFP transgenic zebrafish, a novel compound, 1H-indole-3-carbaldehyde quinolin-8-yl-hydrazone, named Lenaldekar, was identified with the potential to specifically attack T-ALL cells (Ridges et al., 2012). Lenaldekar also has a potential effect against autoimmune diseases such as multiple sclerosis, as they are caused by an off-target activity of T cells (Cusick et al., 2012). Currently there are ongoing clinical trials to study the effectiveness of this promising compound. These examples highlight the power of zebrafish models for screening novel chemical compounds affecting normal, abnormal or malignant hematopoiesis (Shafizadeh et al., 2004; Yeh et al., 2009; Paik et al., 2010; Gutierrez et al., 2014; Arulmozhivarman et al., 2016).

Future studies addressing malignancy heterogeneity may combine chemical screens with scRNAseq to identify therapy-resistant cells and explore the mechanisms underpinning resistance to treatment in individual cells, a fundamental unresolved question in the cancer research field.

### Effects of Perturbations in Embryonic HSC Generation and Adult Hematopoiesis

Several acute myeloid leukemia (AML) predisposition syndromes are caused by innate mutations in transcription factors that affect embryonic hematopoiesis, such as Gata2 and Runx1 (Babushok et al., 2016), suggesting that perturbations in embryonic hematopoiesis affect the adult HSC compartment. As the effects of alterations in embryonic hematopoiesis can be easily monitored in zebrafish throughout development, as well as during adulthood, this is an excellent system to study AML predisposition syndromes. Until the recent development of targeted gene editing, manipulating the zebrafish genome to create specific mutations for making knockout and knockin animals was challenging. Although TILLING (Targeting Induced Local Lesions in Genomes) was a significant advancement, this is a costly method that requires thousands of fish to search for a STOP codon in the gene of interest. Moreover, TILLING is rather limiting as it does not allow to induce specific mutations (Wienholds et al., 2003; Draper et al., 2004). Targeting the zebrafish genome with zinc-finger nucleases was the beginning of a new era in zebrafish biology, as selected genes could finally be specifically targeted for genome editing (Amacher, 2008; Foley et al., 2009). Shortly after this technology was introduced, TALENS (Dahlem et al., 2012; Hwang et al., 2014; Huang et al., 2016; Liu et al., 2016), and more recently, CRISPR/Cas9 (Hruscha et al., 2013; Irion et al., 2014; Shah et al., 2015; Li et al., 2016; Liu et al., 2017) were developed. Whilst it is relatively easy to generate knockouts and large deletions with these gene-targeting techniques, making knockin animals remains challenging. Nevertheless, several laboratories have successfully created knockin animals by using CRISPR/Cas9 and co-injecting a repair template to facilitate homology-directed repair (Hruscha et al., 2013; Auer et al., 2014; Albadri et al., 2017; Kesavan et al., 2017). Additionally, Cre/lox, Flp/FRT, and ΦC31 systems are also currently being used in zebrafish for precise genome editing (Mosimann et al., 2013; Felker and Mosimann, 2016; Carney and Mosimann, 2018). Importantly, tissue-specific expression of Cas9 in the hematopoietic system can be performed in zebrafish to enable conditional manipulation of hematopoietic cells (Ablain et al., 2015). A major caveat in both perturbing the zebrafish genome and comparing the zebrafish with the mammalian transcriptome in the context of clinical translation, is, as previously mentioned, the Teleost genome duplication (Glasauer and Neuhauss, 2014). As a result most genes are present twice with (partially) redundant biological roles. This means that for a complete perturbation of a mammalian gene, the zebrafish counterparts have to be removed both, complicating genetic crossings and analyses.

### Epigenetic Regulation of the Hematopoietic System

Chromatin conformation is essential for controlling gene expression, and deregulation of this process may cause malignant transformation (Groschel et al., 2014). Zebrafish is an excellent system to explore the mechanisms underlying chromatin regulation and to evaluate the effects of chromatin-modifying drugs *in vivo*. Gene regulatory elements can be identified in zebrafish using chromatin immunoprecipitation combined with sequencing (ChIP-seq), however, the technique is limited by the low number of zebrafish-specific antibodies currently available and the large amount of input material required (Havis et al., 2006; Trompouki et al., 2011; Bogdanovic et al., 2013). ChIP-seq has been mostly used in early zebrafish embryos (Paik et al., 2010; Vastenhouw et al., 2010; Bogdanovic et al., 2012; Xu et al., 2012; Winata et al., 2013; Nelson et al., 2017; Meier et al., 2018). Antibodies against histone marks, which are highly conserved between species, have been successfully utilized in zebrafish erythrocytes to describe the potential locus control region (LCR) regulating globin expression (Ganis et al., 2012). Moreover, given the functional conservation of these genes, zebrafish is useful to functionally validate enhancers identified in mouse and/or human models (Tijssen et al., 2011; Chiang et al., 2017).

Other techniques for identifying gene regulatory elements are based on the detection of open chromatin, for instance, assay for transposase-accessible chromatin with high-throughput sequencing (ATAC-seq). ATAC-seq requires much less input material than ChIP-seq, and has even been used successfully with single cells (Fernandez-Minan et al., 2016; Doganli et al., 2017). This method allowed the identification of endothelial enhancers (Quillien et al., 2017) and revealed the role of cohesin in rearranging the genomic architecture during the transition of maternal to zygotic transcription in early embryos (Meier et al., 2018). Combining scRNA-seq with ATAC-seq and immune phenotypic analysis is a powerful approach to integrate our understanding of lineage differentiation with the regulatory elements involved in that process (Buenrostro et al., 2018). DNA methylation studies can also be used to understand chromatin accessibility, although more material is needed in these methods. Methylation experiments have been conducted in zebrafish albeit not specifically in the hematopoietic system (Lee et al., 2015; Kaaij et al., 2016). Since many tissue-specific fluorescent lines exist in zebrafish, future research should aim to identify enhancers and promoters in specific cell types, rather than using whole embryos.

Despite the advantages of ATAC-seq and methylation analyses, these approaches cannot offer the same information as ChIP-seq. Thus, improved ChIP-seq protocols, such as the high sensitivity indexing-first chromatin immunoprecipitation approach (iChIP) developed in Ido Amit’s laboratory, should be adapted to zebrafish (Gury-BenAri et al., 2016). Moreover, it would be important to unravel chromatin interactions in active enhancer and promoter regions during hematopoiesis. However, although chromatin conformation has been studied in early zebrafish embryos (Gomez-Marin et al., 2015; Fernandez-Minan et al., 2016), to date no studies have addressed this question specifically in zebrafish hematopoiesis. It is important to mention the combined effort of many groups to collate all available genome-wide data in zebrafish in the DANIO-CODE Data Coordination Center^1^ (Tan et al., 2016). This recently launched database will provide an easy access to high-quality genome data to all scientists.

### Proteomics and Metabolomics Studies

In the era of genome-wide technology, gene expression studies should be complemented with proteomic studies, as transcriptional and translational outcomes can sometimes differ. Additionally, the extension of these analyses to metabolomics may uncover another layer of regulation critical for hematopoiesis. Indeed, it was recently shown that dormant stem cell populations have low metabolic activity, and this is required to maintain the hematopoietic system during aging and periods of intense stress (Cabezas-Wallscheid et al., 2017). Although proteomics and metabolomics methods have not yet been extensively explored in zebrafish, particularly in the hematopoietic system, some studies have reported differences between transcript and protein levels in multiple genes by using proteomic analyses either in whole zebrafish embryos or in specific cell populations during regeneration (Alli Shaik et al., 2014; Baral et al., 2014; Rabinowitz et al., 2017). Metabolomics has proven useful to understand the neurological damage resulting from chemical perturbations in zebrafish embryos (Ong et al., 2009; Rabinowitz et al., 2017; Roy et al., 2017). Finally, as mass spectrometry analyses are constantly improving, the sensitivity of these methods will likely overcome the current problem of heterogeneous and low cell-number populations.

## Conclusion

**FIGURE 2:**
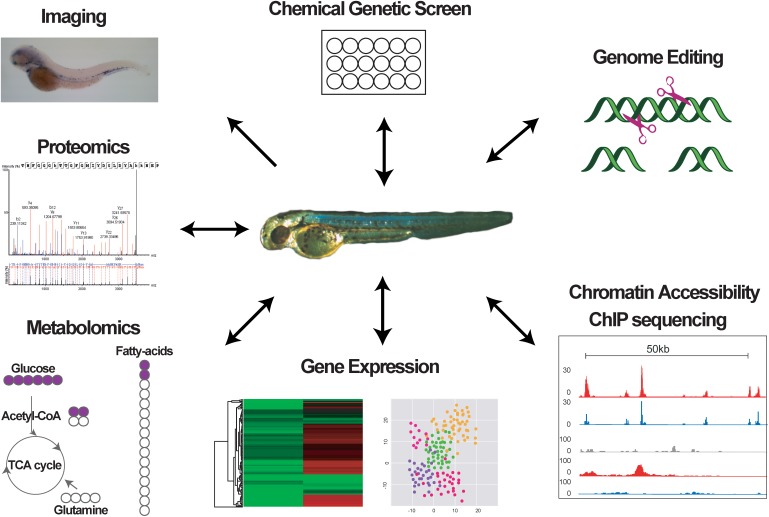
Graph indicating different methods used to study zebrafish hematopoiesis.

The zebrafish has become an invaluable model system for understanding how HSCs form and are maintained, and how hematopoietic cell differentiation is regulated during embryogenesis and in adulthood. The unique advantages offered by this model system over traditional mouse models regarding the use in chemical screens and the accessibility during embryonic stages allowing easy manipulation and visualization and tracing into adult stages, in combination with recent new technologies (**Figure 2**), have opened the way for novel exciting hypotheses on the mechanisms promoting hematopoietic diseases, the role of the niche in normal and malignant hematopoiesis, and the effect of chemical compounds on malignant cells. The high conservation between the zebrafish and human hematopoietic systems means that discoveries in fish may have strong translational potential and important clinical implications for the treatment of hematopoietic diseases.

## Author Contributions

EdP and ET conceived and wrote this manuscript.

## Conflict of Interest Statement

The authors declare that the research was conducted in the absence of any commercial or financial relationships that could be construed as a potential conflict of interest.

## Footnotes

1 https://danio-code.zfin.org
